# Contouring atlas and essential points for radiotherapy in rectal cancer

**DOI:** 10.1093/jrr/rraf013

**Published:** 2025-03-27

**Authors:** Keiko Nemoto Murofushi, Kayoko Tsujino, Yoshinori Ito, Masahiko Okamoto, Hiroshi Doi, Hirofumi Ogawa, Masakatsu Onozawa, Terufumi Kawamoto, Norio Katoh, Keiichi Jingu, Atsuya Takeda, Keiji Nihei, Hirokazu Makishima, Hiroshi Mayahara, Hideya Yamazaki, Hiroshi Igaki

**Affiliations:** Division of Radiation Oncology, Department of Radiology, Tokyo Metropolitan Cancer and Infectious Diseases Center Komagome Hospital, 3-18-22 Honkomagome, Bunkyo-ku, Tokyo, 113-8677, Japan; Department of Radiation Oncology, Hyogo Cancer Center, 13-70 Kitaojicho, Akashi-shi, Hyogo, 673-0021, Japan; Department of Radiation Oncology, Showa University School of Medicine, 1-5-8 Hatanodai, Shinagawa-ku, Tokyo, 142-8666, Japan; Department of Radiation Oncology, Gunma University Graduate School of Medicine, 3-39-22 Showa-machi, Maebashi-shi, Gunma 371-8511, Japan; Department of Radiation Oncology, Kindai University Faculty of Medicine, 377-2, Ohno-Higashi, Osaka-Sayama, Osaka, 589-8511, Japan; Radiation and Proton Therapy Center, Shizuoka Cancer Center, 1007 Shimonagakubo, Nagaizumi-cho, Sunto-gun, Shizuoka 411-8777, Japan; Funabashi Municipal Medical Center, 1-21-1 Kanasugi, Funabashi-shi, Chiba, 273-8588, Japan; Department of Radiation Oncology, Juntendo University Graduate School of Medicine, 2-1-1 Hongo, Bunkyo-ku, Tokyo, 113-8431, Japan; Department of Radiation Oncology, Hokkaido University Faculty of Medicine, N15-W7, Kitaku, Sapporo 060-868, Japan; Department of Radiation Oncology, Tohoku University Graduate School of Medicine, 1-1 Seiryo-machi, Aoba-ku, Sendai, Miyagi 980-8574, Japan; Department of Radiology, Keio University School of Medicine, 35, Shinano, Shinjuku-ku, Tokyo 160-8582, Japan; Department of Radiation Oncology, Faculty of Medicine, Osaka Medical and Pharmaceutical University, 2-7 Daigakumachi, Takatsuki-shi, Osaka 569-8686, Japan; Department of Radiation Oncology, University of Tsukuba Hospital, 2-1-1 Amakubo, Tsubuka, Ibaraki 305-8576, Japan; Division of Radiation Oncology, Kobe Minimally Invasive Cancer Center, 8-5-1, Minatojima-Nakamachi, Chuou-Ku, Kobe, Hyogo 650-0046, Japan; Department of Radiology, Graduate School of Medical Science, Kyoto Prefectural University of Medicine, 465 Kajii-cho, Kawaramachi-Hirokoji, Kamigyo-ku, Kyoto 602-8566, Japan; Department of Radiation Oncology, National Cancer Center Hospital, 5-1-1 Tsukiji, Chuo-ku, Tokyo 104-0045, Japan

**Keywords:** total neoadjuvant therapy, nonoperative management, radiotherapy, mesorectum, atlas

## Abstract

In the last decade, the role of radiotherapy in rectal cancer has changed significantly with the introduction of total neoadjuvant therapy (TNT) and nonoperative management (NOM). For the setting of irradiation field in rectal cancer, the pararectal, lateral lymph nodes, and those along the inferior mesenteric artery (IMA) are most important. In total mesorectal excision (TME), the root of the IMA is dissected. In the atlas of pelvic irradiation for rectal cancer, the setting of the upper margin of the mesorectum varies from atlas to atlas, and no atlas sets the upper margin of the mesorectum to the root of the IMA. In particular, there is no consensus on the definition of anatomical boundaries regarding the lymph nodes along the superior rectal artery (SRA). The upper margin of the irradiation field in clinical trials of preoperative radiotherapy and TNT is generally set at the level of the internal and external iliac artery branches, L5/S1, or S2/S3. However, it is not necessary to include the entire mesorectum to the root of the IMA in patients undergoing preoperative radiotherapy plus TME. Conversely, for patients receiving NOM, the irradiation field may have to include the mesorectum to the IMA root, though the incidence of lymph node metastasis and gastrointestinal adverse events merits consideration. It is increasingly important to determine the extent of clinical target volume around the SRA region and the setting of the upper margin of the irradiation field after formulating the treatment policy together with the surgeons and medical oncologists.

## INTRODUCTION

Preoperative radiotherapy is recommended for rectal cancer patients with a lower tumor (Rb) extending beyond the muscularis propria (T3 or greater) and/or with pelvic lymph node metastasis. The aims of preoperative radiotherapy are mainly to improve local control and avoid permanent colostomy achieved by tumor shrinkage [1–4]. Preoperative radiotherapy reduces pelvic recurrence to 6–9%; however, a high incidence of distant metastasis remains a problem [3, 5]. Therefore, total neoadjuvant therapy (TNT), which includes systemic chemotherapy and radiotherapy before surgery, has been performed to reduce the incidence of distant metastasis and improve the pathological complete response [6–9]. Several prospective clinical trials have evaluated the efficacy of nonoperative management (NOM) in avoiding surgery in patients who achieve clinical CR after radiotherapy. The Organ Preservation for Rectal Adenocarcinoma trial [10], a randomized controlled phase II trial examining the survival and organ preservation rates between the induction chemotherapy group (systemic chemotherapy followed by chemoradiotherapy) and the consolidation chemotherapy group (chemoradiotherapy followed by systemic chemotherapy), showed that a high rate of total mesorectal excision (TME)-free survival (54%) was achieved in the consolidation chemotherapy group. Various strategies have been developed for treating rectal cancer, and the role of radiotherapy has changed significantly. Therefore, it is desirable to set irradiation fields according to the aims and strategies of rectal cancer treatment. For patients treated with preoperative radiotherapy, the irradiation field must be set according to the operative procedure. However, the standard operative procedure differs depending on the country and facility. Furthermore, for patients treated with NOM, it is necessary to include lymph node regions with a high probability of lymph node metastasis in the radiation field because lymph node dissection may not be performed. In this article, we discuss the lymph node region with a high frequency of metastasis and pelvic recurrence sites post-radical surgery for rectal cancer patients. Additionally, revisions in the Japanese Society for Radiation Oncology (JASTRO) guideline (2024 edition) are described with reference to the pelvic lymph node atlas and considerations for setting radiation fields based on recent clinical trials and treatment strategies.

### Regional lymph node dissection and preferred sites of postoperative pelvic recurrence in rectal cancer

Primary tumors in the lower rectum and the most frequently metastasized perirectal lymph nodes are localized within the mesorectum, and the introduction of TME in 1982, in which the entire mesorectum is removed (the upper border is the root of the inferior mesenteric artery; IMA), has reduced the incidence of local recurrence [11]. Currently, the standard techniques are TME or tumor-specific mesorectal excision (ME), which involve excision of 3 cm caudal to the mesorectum from the caudal border of the tumor for tumors cephalic to the peritoneal reflection and up to 2 cm caudal to the mesorectum for those caudal to the peritoneal reflection [12]. In a study on lymph node metastasis frequency in rectal cancer patients [13], pararectal lymph node metastasis within the mesorectum occurred in 46% of cases, lymph nodes along the IMA (inferior mesenteric trunk and inferior mesenteric root nodes) in 28%, and lateral lymph nodes in 13%. For patients with stage I–III lower rectal cancer, the incidences of inferior mesenteric trunk and inferior mesenteric root lymph node metastasis were 12.3% and 0.7%, respectively [14]. Moreover, in terms of the characteristics of lymph node metastasis according to the location of the primary tumor, while upper rectal cancer metastasizes to lymph nodes along the IMA, lower rectal cancer metastasizes to multidirectional lymph nodes with a higher frequency of lateral lymph node metastasis in addition to pararectal lymph nodes and lymph nodes along the IMA. The Japanese Classification of Colorectal, Appendiceal, and Anal Carcinoma recommends dissection of the pararectal lymph nodes, lymph nodes along the IMA, and lateral lymph nodes as regional lymph nodes in radical surgery for lower rectal cancer [15]. In Europe and the United States, TME alone is the standard technique because preoperative radiotherapy is performed instead of lateral node dissection (lateral dissection) [16]. Notably, lateral lymph node dissection (LLND) can cause urinary and sexual dysfunctions because of its proximity to the pelvic autonomic nervous system. The guidelines of the Japanese Society for Cancer of the Colon and Rectum strongly recommended lateral dissection in patients with lower rectal cancer and lateral lymph node metastasis at preoperative or intraoperative diagnosis [12]. Regarding the significance of lateral dissection in patients without obvious lateral lymph node metastasis, the Japan Clinical Oncology Group (JCOG) 0212 trial evaluated the non-inferiority of recurrence-free survival as the primary endpoint in the ME group to the ME + LLND group for the lower rectal cancer patients without lateral lymph nodes larger than 10 mm in short diameter on preoperative computed tomography or magnetic resonance imaging [17–19]. The results indicated that the hazard ratio for recurrence-free survival between the ME group and the ME + LLND group was 1.07 (90.9% confidence interval: 0.86–1.36). Furthermore, the non-inferiority of the ME group compared to the ME + LLND group in the JCOG 0212 trial, with the non-inferiority margin's upper limit set at 1.34, was not statistically significant (p_non-inferiority_ = 0.0547). The incidence of pelvic recurrence was significantly higher in the ME group than in the ME + LLND group; however, the incidence of central pelvic recurrence showed no difference. Meanwhile, the ME group exhibited a significantly higher incidence of lateral pelvic recurrence. Based on the above considerations, the guideline reported by the Japanese Society for Cancer of the Colon and Rectum weakly recommended that LLND be performed if lateral lymph node metastasis is negative preoperatively or intraoperatively because it is expected to reduce lateral pelvic recurrence, although the survival benefit of LLND is limited [12]. At present, a few randomized controlled trials have reported that an equivalent therapeutic effect is obtained between preoperative radiotherapy and LLND [20], and the criteria for patients with lower rectal cancer for whom LLND can be omitted are not clear. The JCOG 0212 trial showed that the incidence of central pelvic recurrence in the ME + LLND group was not significantly lower than that in the ME group; thus, preoperative radiotherapy is expected to reduce the incidence of pelvic recurrence, especially central pelvic recurrence. The ongoing JCOG 2207 trial evaluated the superiority of overall survival in TME + selective LLND after total neoadjuvant therapy, including systemic chemotherapy and radiotherapy, over TME + LLND followed by systemic chemotherapy as the standard therapy.

On the other hand, the report examining local recurrence after radical surgery classified pelvic sites into four categories: posterior (posterior pelvic subsite), lateral (lateral pelvic subsite), inferior (inferior pelvic subsite), and anterior (anterior pelvic subsite), showing that 49% of local recurrence sites were posterior (e.g. anterior sacrum), 21% were lateral, 12% were inferior (e.g. perineum) and 17% were anterior (e.g. near the bladder) [13].

These reports suggest that the primary tumor and pararectal lymph nodes within the mesorectum, anterior sacrum, lateral lymph nodes, and lymph nodes along the IMA are important target regions for radiotherapy in patients with lower rectal cancer.

**Table 1:** Prophylactic lymph node area in clinical trials

Clinical trial	Treatment	Technique	Lymph node region	Superior border of the irradiation field or CTV
Swedish Rectal Cancer Trial [1]	Surgery vs. preoperative RT + surgery	2D	Pararectal (± lymph node along IMA), presacral, and internal iliac lymph node	L4/5 (superior border of the irradiation field)
Dutch Colorectal Cancer Group [2]	TME vs. preoperative RT + TME	2D	Pararectal, presacral, and internal iliac lymph node	L5/S1 (superior border of the irradiation field)
German Rectal Cancer Study Group [3]	Preoperative RT + TME vs. preoperative CRT + TME	2D (3D)	Pararectal lymph node	S2/3 or 3 cm cephalad to the tumor (superior border of CTV)
EORTC22921 [5]	Preoperative RT + TME ± adjuvant CTx vs. preoperative CRT + TME ± adjuvant CTx	2D (3D)	Pararectal lymph node	S2/3 or 3 cm cephalad to the tumor (superior border of CTV)
RTOG0822 [20]	Preoperative CRT + TME + adjuvant CTx	IMRT	Pararectal, presacral, internal iliac and obturator lymph node (T4: external iliac lymph node)	The bifurcation of internal and external iliac artery (superior border of CTV)
RAPIDO [7]	Preoperative CRT + TME + adjuvant CTx vs. preoperative RT + CTx + TME	3D/IMRT	Pararectal, presacral and ± internal iliac lymph node, (T4 invading to bladder, prostate, and cervix and vagina: external iliac lymph node)	The bifurcation of internal and external iliac artery. If the bifurcation exceeds the level of L5/S1, S2/3 (superior border of CTV)

CTV, clinical target volume; RT, radiotherapy; TME, total mesorectal excision; CRT, chemoradiotherapy; CTx, chemotherapy; IMA, inferior mesenteric artery.

### Prophylactic lymph node region in clinical trials of perioperative radiotherapy for rectal cancer

Most clinical trials of perioperative radiotherapy for rectal cancer have mainly included patients with primary tumors in the upper rectum (Ra) and lower rectum (Rb), although some have included the anal canal (P) [1–3, 6, 21–26]; furthermore, the prophylactic pelvic lymph node regions to be included in the radiation field vary depending on the clinical trial [1–3, 6, 21, 22]. Table 1 shows the lymph node regions included in the radiation fields of representative clinical trials. The Dutch Colorectal Cancer Group trial included the same pelvic lymph node regions as those of the Swedish Rectal Cancer Trial; however, the superior margin of the radiation field was set at a promontory [2, 25]. The results showed a significantly favorable local control rate in the preoperative radiotherapy group but no difference in the overall survival rate compared with that of the surgery-alone group. The EORTC22921 trial, which showed the benefit of preoperative chemoradiotherapy, included pararectal lymph nodes but no lateral lymph nodes, and the superior margin of the radiation field was set to S2/3 or 3 cm cranial to the tumor [21]. The RAPIDO trial, which demonstrated the benefit of TNT with preoperative radiotherapy (25 Gy/5 fractions) followed by systemic chemotherapy [6], included the pararectal lymph node, presacral, internal iliac, and obturator lymph nodes to the clinical target volume (CTV), with the superior margin being the level of the internal and external iliac artery branches, or the S1/2 level if the level of such branches exceeds L5/S1. As mentioned previously, the radiation field settings vary depending on the clinical trial, but most include the pararectal, presacral, and internal iliac lymph nodes (plus obturator lymph nodes). In the Swedish Rectal Cancer trial [1, 23], Dutch Colorectal Cancer Group trial [2, 25], and EORTC22921 [21], a part of the obturator lymph node region was included in the radiation field because the radiation fields were set up with bone structures and anterior organs as the Merkmal. The external iliac lymph nodes are not considered important regions for preoperative radiotherapy in rectal cancer. However, recent protocols for clinical trials have stated that the external iliac lymph nodes are included in the CTV for patients with T4 involving the anterior organ [24, 26]. There are several discussions on whether inguinal lymph nodes should be included in the CTV. Notably, the RAPIDO trial indicated that inguinal lymph nodes were included in the CTV when the primary tumor invaded the anal canal. However, the proportion of patients in which inguinal lymph nodes are encompassed within the CTV is unknown. [6]. Therefore, the inclusion of the external iliac lymph nodes in the CTV for patients with T4 should be considered at each institution and for each individual patient.

### Atlas of pelvic lymph node regions in radiotherapy for rectal cancer

Atlas of pelvic lymph node regions in radiotherapy for rectal cancer was reported by the Radiation Therapy Oncology Group (RTOG) [27], the Australasian Gastrointestinal Trials Group (AGITG) [28], and the principal radiation oncology society [29], including the European Society of Radiotherapy & Oncology, the American Society for Radiation Oncology, the Tasman Radiation Oncology Group, and the European Organization for Research and Treatment of Cancer. Accurate contouring of the primary tumor, lymph node regions, and normal organs is crucial in intensity-modulated radiation therapy (IMRT) for rectal and anal canal cancers. The RTOG atlas, determined by nine radiation oncologists, provided contouring recommendations for lymph node regions and normal organs. Meanwhile, the AGITG atlas, initiated in 2010 by radiation oncologists, offered recommendations for the primary tumor, lymph node regions, and normal organs. The international consensus guidelines, authored by the principal radiation oncology society, presented contouring recommendations for lymph node regions in radiotherapy targeting rectal cancer. The contouring atlas for rectal cancer, aligned with the JASTRO guidelines, was developed based on these recommendations.

### Clinical target volume for prophylactic nodal regions

**Fig. 1:**
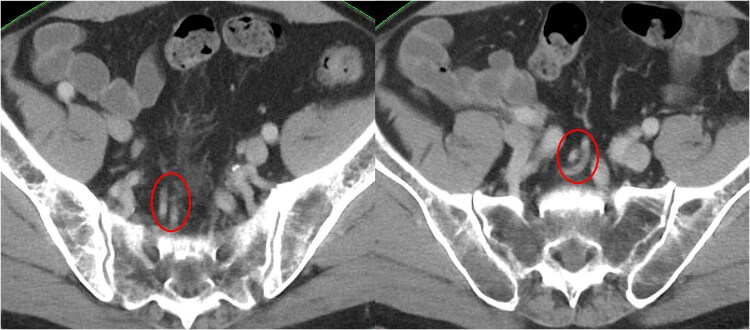
Lymph node along the superior rectal artery. The superior rectal artery is represented by the circle.

In the atlas recommended by the RTOG [27], the CTV for the prophylactic lymph node regions was described in three categories: the CTVA included the internal iliac, presacral lymph nodes, and mesorectum; the CTVB included the external iliac lymph nodes; and the CTVC included the inguinal lymph nodes. For patients with rectal cancer, the CTVA should be considered. For those with T4 tumors involving the uterus, vagina, bladder, or prostate, the CTVB is appropriate; finally, the CTVC is necessary for tumors invading the anal verge, perineal skin, or the lower one-third of the vagina, though not required. The atlas of the CTV describes the division of the CTVA into the lower, middle, and upper pelvis; however, there is no detailed description of the six directional boundaries of the CTVA-C: cephalocaudal, ventral-dorsal, left, and right. In contrast, the atlas recommended by the AGITG divides the lymph nodes into the following seven regions [28]: i) mesorectum, ii) presacral lymph nodes, iii) internal iliac lymph nodes, iv) external iliac lymph nodes, v) obturator lymph nodes, vi) ischiorectal fossa, and vii) inguinal lymph nodes. For these seven regions, the 6-directional boundaries are defined. The international consensus guidelines classify the lymph nodes into seven regions: i) mesorectum, ii) presacral lymph nodes, iii) lateral lymph nodes, iv) external iliac lymph nodes, v) ischiorectal fossa, vi) inguinal lymph nodes, and vii) sphincter complex. For the first six regions (i–vi), 6-directional boundaries are specified. For each lymph node region, 6-directional boundaries are specified (Table 2). The RTOG and AGITG atlases identify the cephalic border of the mesorectum at the rectosigmoid junction level, while the international consensus guidelines [29] describe it as the bifurcation of the superior rectal artery (SRA) or sigmoid colon artery into the IMA. Further, the cephalic border of the mesorectum was defined as the root of the IMA according to the Japanese Classification of Colorectal, Appendiceal, and Anal Carcinomas [15]. The mesorectum, situated along the SRA cephalad of the rectosigmoid junction (Fig. 1), is classified within the presacral lymph node region according to the AGITG atlas, described as ‘including presacral vessels.’ The RTOG atlas did not describe the lymph nodes along the SRA, and the definition of the mesorectum varied from atlas to atlas. The mesorectum is the most important region in rectal cancer because of the high frequency of lymph node metastasis. However, the upper region is easily dissected surgically, and including the upper region in the radiation field may increase the volume of the irradiated small bowel, which may lead to an increase in adverse events. The superior border of the mesorectum, defined in the Japanese Classification of Colorectal, Appendiceal, and Anal Carcinoma and international guidelines, can be more cephalad than the superior border of the irradiated field in clinical trials of perioperative radiation therapy for rectal cancer. The lymph nodes along the IMA are relatively highly metastatic lymph node regions with a high incidence of distant metastasis; however, improved survival can be achieved by dissecting the root of the IMA [30]. If radical surgery is performed after radiation therapy as a neoadjuvant therapy, rather than aiming for organ preservation in NOM, there is little need to include the lymph nodes along the IMA in the radiation field, considering that the mesorectum is always dissected. On the other hand, when surgical treatment is not performed because of NOM, it is necessary to consider including the lymph nodes along the IMA in the irradiation field. However, while the incidence of metastasis in inferior mesenteric trunk lymph nodes exceeded 10%, inferior mesenteric root lymph nodes were rarely metastasized [14]. At present, the definition of the upper border of the CTV has not been fully explored in NOM.

**Table 2:** Anatomical boundaries of the pelvic and inguinal lymph nodes of the Japanese Society for Radiation Oncology guidelines

	CTV-Rec				CTV-Ext	CTV-Ing
	Mesorectum	Presacral LN	Internal iliac LN	Obturator LN	External iliac LN	Inguinal LN
Cranial	The recto-sigmoid junction	The sacral promontory (L5/S1)	Bifurcation of the external and internal iliac arteries (L5/S1).	Three to 5 mm cranial to the obturator canal.	Bifurcation of the external and internal iliac arteries (L5/S1).	The level of the external iliac artery where it transitions to the femoral artery (the superior pubic rami).
Caudal	The ano-rectal junction, or where the levator muscles fuse with the external sphincter muscles	The inferior edge of the coccyx	The level of the obturator canal, or at the level where there is no space between the obturator internus muscle and bladder/seminal vesicle.	The obturator canal.	The level of the external iliac artery where it transitions to the femoral artery (the superior pubic rami).	The lower edge of the ischial tuberosities, or 20 mm caudal to the junction of the saphenous vein and femoral vein.
Anterior	For males; bladder, seminal vesicle, prostate, and penile bulb. For females; bladder, uterus, and vagina. Dorsal 10 mm of the bladder, seminal vesicle, and uterus is added as an internal margin.	Ten mm anterior to the anterior sacral border, including superior rectal vessels.	The upper pelvis; 7 mm around the margin to the internal iliac vessels. The lower pelvis; the obturator internus muscle or bone.	The anterior extent of the obturator internus muscle.	Seven mm around the margin to the external iliac vessels.	Subcutaneous 5 mm (consider including the skin surface if there is a risk of skin invasion or if the subcutaneous fat is thin).
Posterior	The presacral space	The anterior border of the sacral bone, including the sacral hollows.		The anterior extent of the internal iliac lymph node region.	The anterior extent of the internal iliac lymph node region.	The femoral triangle formed by the iliopsoas, pectineus, and adductor longus muscles.
Lateral	The upper pelvis; the medial border of the internal iliac lymph node. The lower pelvis; medial edge of levator ani.	The sacro-iliac joints.	The upper pelvis; iliopsoas muscle. The lower pelvis; the obturator internus muscle or bone.	The obturator internus muscle.	The iliopsoas muscle.	The medial border of the suture and psoas muscle.
Medial			The upper pelvis; 7 mm around the margin to the internal iliac vessels. The lower pelvis; the mesorectum and presacral space.	Bladder	Bladder, otherwise 7 mm around the margin to the external iliac vessels.	The lateral border of the long adductor muscle or medial border of the puborectalis muscle.

CTV, clinical target volume; Rec, rectum; Ex, external iliac lymph node; Ing, inguinal lymph node; LN, lymph node.

### Clinical target volume for prophylactic nodal regions in the Japanese Society for Radiation Oncology guideline and revisions in the Japanese Society for Radiation Oncology guideline 2024 edition

**Fig. 2:**
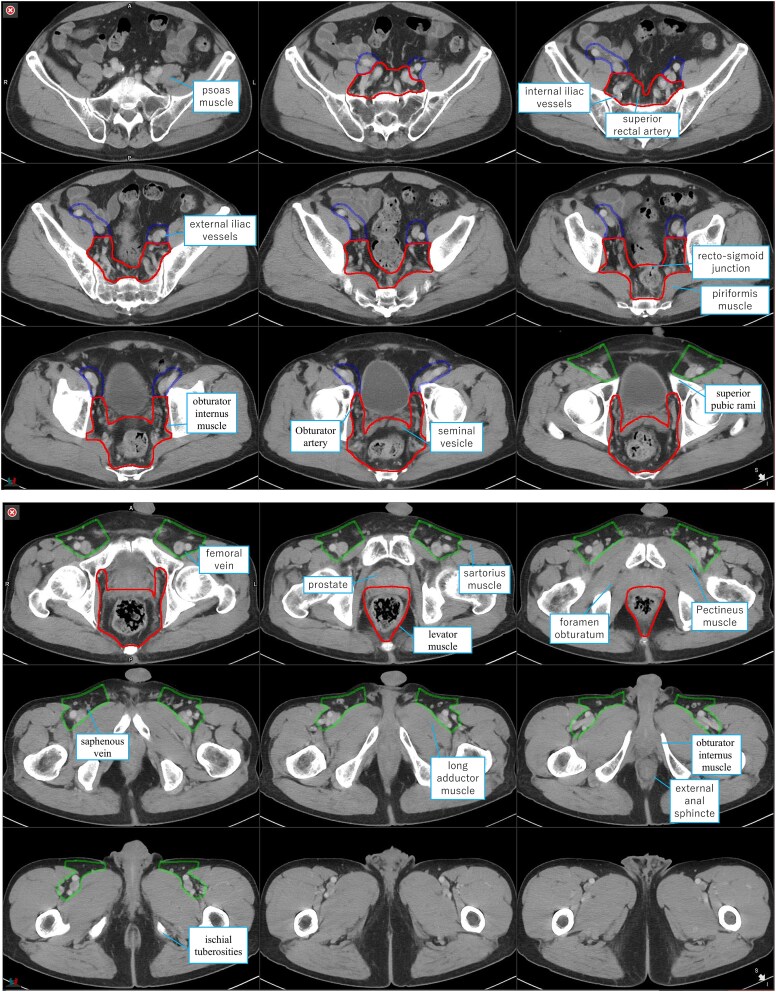
The contouring atlas for rectal cancer in accordance with the Japanese Society for Radiation Oncology guidelines. The red line represents CTV-Rec (mesorectum, presacral, internal iliac, and obturator lymph nodes), the blue line represents CTV-Ex (external iliac lymph node), and the green line represents CTV-Ing (inguinal lymph node). CTV, clinical target volume.

The JASTRO guideline categorizes the CTV into three groups for the prophylactic lymph node regions, aligning with the RTOG guideline: CTV-Rec (mesorectum, presacral, internal iliac, and obturator lymph nodes), CTV-Ext (external iliac lymph node), and CTV-Ing (inguinal lymph node) (Fig. 2). Lymph nodes situated in the cephalad of the rectosigmoid junction along the SRA are included within the presacral lymph node region, described as ‘including superior rectal vessels’ (Table 2). The 2024 edition of the JASTRO guideline introduces statements noting that while the mesorectum harbors key lymph nodes with a high incidence of metastasis, the metastatic frequency diminishes as the lymph nodes become more cephalic. Specifically, the frequency of lymph node metastases decreases sequentially from the pararectal lymph nodes, to the inferior mesenteric trunk, and finally to the inferior mesenteric root nodes. Given the mesorectum's surgical accessibility, the determination of the CTV's extent near the SRA and the setting of the superior margin should be conducted collaboratively with surgeons and medical oncologists, reflecting the overarching treatment strategy. Concerning the anatomical boundaries of inguinal lymph nodes, recent studies [31, 32] reveal that atlases by AGITG and RTOG might not comprehensively cover lymph node metastases in the anterior and medial regions. Consequently, revisions have been made based on these findings [31, 32], extending the boundaries to subcutaneous 5 mm (including the skin surface if there is a risk of skin invasion or if subcutaneous fat is scant) and to the lateral border of the long adductor muscle or the medial border of the puborectalis muscle, respectively.

### The benefit of intensity-modulated radiotherapy in perioperative radiotherapy of rectal cancer

In the RTOG0822 trial, which evaluated the benefits of IMRT, the incidence of grade 2–5 acute gastrointestinal adverse events was compared between the RTOG0822 trial (concurrent use of capecitabine + oxaliplatin [CAPOX] with pelvic IMRT administered at 45 Gy in 25 fractions, followed by additional irradiation with three-dimensional conformal radiation therapy [3D-CRT] administered at 5.4 Gy in three fractions) and the RTOG0247 trial (concurrent use of capecitabine + oxaliplatin with pelvic 3D-CRT followed by additional radiation with 3D-CRT) [33]. This incidence was 40% in the RTOG0247 trial and 51.5% in the RTOG0822 trial (*P* = 0.93), indicating that IMRT did not cause any adverse effects. This retrospective study compared the acute and late adverse events between 3D-CRT and IMRT in preoperative chemoradiotherapy for rectal cancer [34]. The pararectal (± a part of lymph node along the IMA), presacral, and internal iliac lymph nodes were included in the CTV, and the incidence of grade 3 or higher acute adverse events was 20% for 3D-CRT and 5% for IMRT, with IMRT showing significantly lower rates (*P* = 0.0081). Similarly, the incidence of late adverse events was lower in the IMRT group compared to the 3D-CRT group (22% vs. 6%; *P* = 0.0039). However, the advantages of IMRT in preoperative radiotherapy for rectal cancer have not been established across multiple trials, and there is no conclusive evidence to strongly recommend IMRT use. In recent years, TNT has been recommended for locally advanced lower rectal cancer. In the RAPIDO trial, 17% of the TNT group who received pelvic irradiation administered at 25 Gy in five fractions followed by six courses of CAPOX or nine courses of fluorouracil + leucovorin + oxaliplatin had grade 3 or higher diarrhea. However, the difference was not significantly significant (10% in the preoperative chemoradiotherapy group) [6]. In a phase II study of NOM in Japan, 23.3% of patients had grade 3 or higher diarrhea after six courses of CAPOX at 25 Gy in five fractions [35]. Therefore, it is necessary to develop a radiotherapy technique that can reduce the incidence of diarrhea.

In the context of radiotherapy for rectal cancer, techniques such as 3D-CRT and IMRT utilize the same CTV for prophylactic lymph node regions, allowing for a shared atlas. Despite the frequent occurrence of metastases to lymph nodes along the SRA, some radiation oncologists may not be well-acquainted with this area. If the CTV excludes the lymph nodes along the SRA, using 3D-CRT will not typically lead to under-dosing; however, IMRT may result in a reduced dose.

## CONCLUSION

Preoperative radiotherapy plays a major role in reducing the incidence of pelvic recurrence in patients with rectal cancer. Recently, the role of radiotherapy has expanded with the introduction of TNT and the ability to avoid surgery by using NOM. The setting of the radiation field should be determined by considering the frequency of lymph node metastasis and the preferred site of recurrence, whereas that of the superior margin of the radiation field should be based on the primary site and the treatment strategy.
